# Pharmaceuticals in the Aquatic Environment: A Review on Eco-Toxicology and the Remediation Potential of Algae

**DOI:** 10.3390/ijerph19137717

**Published:** 2022-06-23

**Authors:** Monika Hejna, Dominika Kapuścińska, Anna Aksmann

**Affiliations:** Department of Plant Physiology and Biotechnology, Faculty of Biology, University of Gdansk, Wita Stwosza 59, 80-308 Gdansk, Poland; monika.hejna@ug.edu.pl (M.H.); dominika.kapuscinska@phdstud.ug.edu.pl (D.K.)

**Keywords:** phycoremediation, contaminants of emerging concern, pharmaceuticals, non-steroidal anti-inflammatory drugs, ecotoxicology, wastewater treatment

## Abstract

The pollution of the aquatic environment has become a worldwide problem. The widespread use of pesticides, heavy metals and pharmaceuticals through anthropogenic activities has increased the emission of such contaminants into wastewater. Pharmaceuticals constitute a significant class of aquatic contaminants and can seriously threaten the health of non-target organisms. No strict legal regulations on the consumption and release of pharmaceuticals into water bodies have been implemented on a global scale. Different conventional wastewater treatments are not well-designed to remove emerging contaminants from wastewater with high efficiency. Therefore, particular attention has been paid to the phycoremediation technique, which seems to be a promising choice as a low-cost and environment-friendly wastewater treatment. This technique uses macro- or micro-algae for the removal or biotransformation of pollutants and is constantly being developed to cope with the issue of wastewater contamination. The aims of this review are: (i) to examine the occurrence of pharmaceuticals in water, and their toxicity on non-target organisms and to describe the inefficient conventional wastewater treatments; (ii) present cost-efficient algal-based techniques of contamination removal; (iii) to characterize types of algae cultivation systems; and (iv) to describe the challenges and advantages of phycoremediation.

## 1. Introduction

The pollution of the aquatic environment has become a worldwide problem, attracting public attention and forcing scientists and governments to enhance their efforts to prevent further degradation of the environment. A variety of pollutants, such as pesticides, heavy metals, polycyclic aromatic hydrocarbons and, more recently, microplastic particles and pharmaceuticals, enter water bodies through anthropogenic activities and threaten the health of plants, animals and humans due to their acute toxicity and potential accumulation risk (chronic effects) [1,2]. Thus, chemical, physical, and biological remediation methods are constantly being developed to deal with this problem. Among them, phycoremediation (remediation using macro- and micro-algae) seems to be a promising choice as a low-cost, environment-friendly and sustainable method. Phycoremediation has already been demonstrated to be useful for heavy metal removal from wastewater [3,4,5,6,7,8,9,10,11,12,13], and now, the potential of algae to remove other anthropogenic contaminants, such as pharmaceuticals, is being intensively studied.

Pharmaceuticals are designed to have a specific beneficial mode of action in humans or animals and represent any chemical product with a biologically active compound that is used for: (i) the diagnosis, treatment or prevention of disease or any health condition in human medicine; (ii) the enhancement of skin health in the beauty care industry; or (iii) the control of enteric diseases, for the increment of the growth performances of livestock and to maintain profitability and sustainability in the agriculture industry [14,15,16]. There are a large number of pharmaceuticals, with more than 3000 used pharmaceuticals registered in the European Union alone [17]. Among different classes of pharmaceuticals, e.g., antibiotics, medicines regulating lipid metabolism, hormonal agents, anti-epileptic drugs and β-blockers, non-steroidal anti-inflammatory drugs (NSAIDs) are the most common drugs used to reduce the inflammation process and relieve pain due to their anti-inflammatory, analgesic and antipyretic properties [18]. Large amounts of these substances are consumed daily due to their wide availability in various non-prescription pharmaceutical formulas [19]. The pharmaceutical industry is one of the most important and continuously growing sectors worldwide, with increasing sales over the last decade [20]. From 2001 to 2003, the annual consumption of ibuprofen corresponded to 128 tons year^−1^ in Germany, 180 tons year^−1^ in Canada and 276 tons year^−1^ in Spain. The annual consumption of amoxicillin antibiotics in 2001 was 110 tons year^−1^ in Germany and Italy. Moreover, approximately 23,000 tons of antibiotics are used every year in the United States of America (USA) [20,21]. The increasing contamination of the environment with pharmaceuticals is not only due to their increasing consumption but also results from inefficiencies in the removal of these compounds using conventional wastewater treatments, which fail to fully remove many pharmaceutical compounds [22,23].

Considering this, pharmaceuticals originating from human activities may simply enter the aquatic environment, mainly via wastewater, and reach different surface water bodies such as streams, rivers, lakes, wetlands, reservoirs, creeks and oceans, as well as ground and drinking water reservoirs [24,25]. Therefore, undigested pharmaceuticals and their metabolites, which constitute a significant class of potentially hazardous aquatic pollutants with no official regulatory standards, can significantly threaten food chains and should be continuously monitored in the environment [26,27]. Unfortunately, there are limited publications relating to pharmaceutical contaminants, and those that do exist often report incomplete and contradictory data. Thus, a review providing an overview of sources, a summary and a critical evaluation of the current knowledge of pharmaceuticals in the environment, as well as possible remediation methods, is needed. Therefore, the aims of this review are: (i) to examine the occurrence of pharmaceuticals as contaminants of emerging concern in wastewater, surface water and drinking water and their toxicity on non-target organisms globally, and to describe the conventional wastewater treatments that are mostly inefficient to cope with pharmaceutical contaminants; (ii) to present different algal-based techniques for contaminant removal as cost-efficient substitutes for conventional wastewater treatments; (iii) to characterize different types of algae cultivation systems and factors which may greatly influence the removal efficiency of contaminants; and (iv) to describe the challenges and advantages of phycoremediation.

## 2. Pharmaceuticals as Contaminants of Emerging Concern

The widespread use of pharmaceuticals causes their continuous emission into the environment [28]. The largest and major sources of pharmaceutical contamination of wastewater globally are (i) urban domestic effluents, (ii) hospital effluents, (iii) animal farming, with animal excretion of pharmaceuticals and their metabolites, and (iv) pharmaceutical manufacturing [29]. Hospitals and medical clinics contribute a significant load of pharmaceuticals into wastewater effluent from medicine excretion by patients and from laboratory, diagnostic and research activities. The total concentration of pharmaceuticals in urban wastewater results from the combination of many factors, such as administrated quantity, excreted percentage and chemical characteristics of the specific compound [20]. Veterinary drug applications in farming animals, inappropriate disposal of unused medicines and fed ingredients containing different pharmaceuticals are the main routes of entry to terrestrial farmlands. Antibiotic use in agriculture is restricted and regulated in many countries [30,31,32]; however, antibiotics can still be used to treat animal infections through veterinary prescriptions [31]. Pharmaceutical manufacturing units are of special concern due to extremely high efflux concentrations with point source contamination, especially in developing countries where proper industrial effluent treatment is lacking [33].

Following the entry of pharmaceuticals into wastewater from the aforementioned sources, they proceed further into environmental matrices including surface water (inland water bodies and seas), groundwater, sediments, soil and even drinking water supplies [24,25,28,34,35,36]. The occurrence of pharmaceuticals in water bodies varies among locations and depends on drug consumption patterns [37]. Concentrations of different pharmaceuticals in drinking water are lower than their therapeutic doses, except for the concentration of ibuprofen in the USA (5850.0 ng/L) and Taiwan (836.7 ng/L); however, the long-term effects of such doses are still not well described (Table 1) [38]. Moreover, a high percentage of consumed pharmaceuticals, for example, NSAIDs, enter sewage sludge and wastewater in all areas (Table 1). The concentration values for all NSAIDs are higher than 200 ng/L in most locations for wastewater (especially for ibuprofen). In Poland, high concentrations of ibuprofen, diclofenac, naproxen and ketoprofen were observed for wastewater (31,250.0 ng/L, 40,570.2 ng/L, 551,960.0 ng/L, 233,630.0 ng/l, respectively; Table 1). This may suggest that the problem of these pharmaceuticals in aquatic bodies is even more common; thus, more studies are required to monitor their exact concentrations and to examine the migration of this group to aquifers. To date, non-steroidal anti-inflammatory drugs are the most frequent pharmaceutical class in Europe, and the highest concentrations of antibiotics have been recorded in Asia [39]. Additionally, the concentrations of different pharmaceuticals in developing countries are higher and less monitored compared with highly developed countries [40]. The consumption of pharmaceuticals also depends on seasonal disease peaks. Antibiotic consumption due to infections results in increased influxes into water and wastewater during the winter and autumn seasons. Similarly, antihistamine influxes increase seasonally with the release of pollen and subsequent allergy treatment [41]. The recent pandemic situation has also increased pharmaceutical use, resulting in a sharp increase in drug loads in water bodies. However, scarce data are available on this due to the pandemic’s unpredictability and unexpected pandemic variations.

Undigested pharmaceuticals and their metabolites constitute a new and significant class of aquatic pollutants and result in a serious threat to the food chain [27,28,64,65,66]. Pharmaceuticals also represent an environmental risk due to the significant effects they can have on a range of non-target aquatic organisms with similar biological functions and receptors [67]. The risk of these substances in water is directly associated with their intact form, but parent compounds can be further transformed by biotic and abiotic processes (microbiological transformation, hydrolysis or photolysis) either in natural water reservoirs or during sewage treatment [68,69]. Products of such transformations may have similar or even higher toxicity compared with the parent compounds, so the influence of these on non-target organisms should also be considered. Accordingly, intact pharmaceuticals and their derivatives may be toxic to animals, plants and algae species, and they may accumulate in their tissues [70,71].

Research has shown that pharmaceuticals not only exhibit short-term (acute) toxicity, but long-term (chronic) exposure should also be considered. Acute toxicity is the effect induced by either a single exposure or multiple exposures in a short time period and often appears as a lethal endpoint (mortality or immobilization). Chronic toxicity is the onset of adverse effects resulting from prolonged and repeated exposure to stressors, which usually appears as a sub-lethal endpoint (growth inhibition, molecular or biochemical alterations or behavioral changes) [1]. The most common chronic toxic effects of pharmaceuticals in non-target animal species are related to (i) locomotive disorders, (ii) endocrine disruption, (iii) genotoxicity, (iv) reproduction disorders, (v) oxidative stress, (vi) body deformations, (vii) teratogenic effects and (viii) reductions in overall organism condition (vitality) (Table 2) [2]. The scale of pharmaceutical toxicity on non-target aquatic organisms is high; however, more research on aquatic animal species has been conducted compared to aquatic plant species (Table 2). Moreover, as trophic levels increase in a food chain, the accumulation of toxins is expected to increase. However, only limited information regarding the propagation of the effects of pharmaceuticals from the lowest to the highest levels of biological organization and their effects at cellular and tissue levels in freshwater species has been reported. Sublethal effects underlined by short- and mid-term exposures may also be alarming, since non-target species are exposed to measurable pharmaceutical concentrations throughout their life [1].

No strict legal regulations on the production, consumption and release of pharmaceuticals into the environment have been implemented on a global scale [39,120]. Such substances are not a target of the monitoring process; however, they have significant potential to damage the aquatic environment [121]. These substances and their metabolites do not have acceptable concentration standards, and their toxicity mechanisms have not been well defined; thus, pharmaceuticals have been classified as contaminants of emerging concern [122,123,124,125]. Emerging contaminants are unregulated pollutants that may be candidates for future legislation depending on the monitoring results of their occurrence or their potential health effects. Therefore, in the near future, legal limits for the concentrations of pharmaceuticals in discharges from wastewater treatment plants that are mainly introduced into the environment will be created [126]. Emerging contaminant substances include many groups of compounds such as pharmaceuticals and personal care products, gasoline additives, endocrine disruptors and illicit drugs [127]. Therefore, pharmaceuticals are potentially hazardous substances with no regulatory standards that have been detected in the environment and natural streams for a relatively short period. As such, these should be monitored due to their potentially toxic impacts on non-target organisms [26]. However, monitoring of pharmaceuticals may be complicated due to their various active chemical structures, the diversified influences they have on living organisms and lacking environmental toxicity data [128,129].

## 3. Conventional Wastewater Treatment and Pharmaceutical Removal Methods

In recent times, research focuses on the increasing presence and negative impacts of toxic substances in aquatic ecosystems; thus, much effort is needed to increase wastewater treatment efficiency and to enhance water use efficiency [130]. To remediate wastewater from toxic substances, conventional wastewater treatment techniques which involve chemical, physical and biological approaches are widely applied. The efficiency of pollutant removal depends on the process used and the type of pollution [131]. The most commonly used chemical methods involve ion exchange, neutralization, calcination, precipitation and reduction. Physical treatments include adsorption, filtration, flocculation, dialysis, electrodialysis, evaporation, reverse osmosis, sedimentation and stream stripping. Biological treatments of contaminated water involve anaerobic digestion, activated sludge, aerated lagoons, waste stabilization and biodegradation by microbial cultures. In biological and chemical methods, the pharmaceuticals are chemically modified to form new degradation products or metabolites; in contrast, most physical processes convert pharmaceuticals from an aquatic to a solid phase [39].

**Figure 1:**
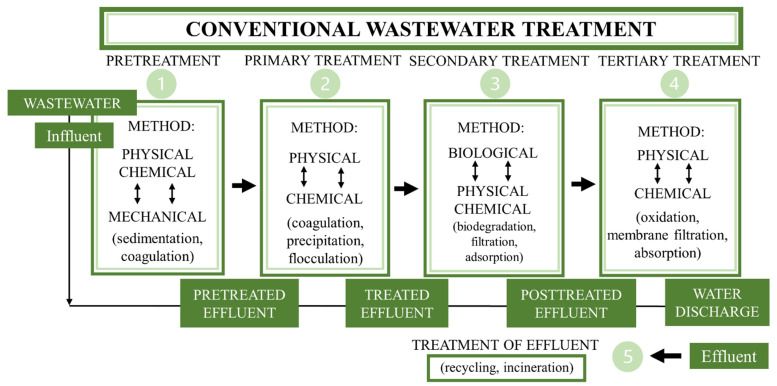
Main steps of conventional wastewater treatment, based on [134].

Conventional wastewater treatments include a combination of the abovementioned processes, which are divided into preliminary, primary, secondary and tertiary wastewater treatments (Figure 1) [39]. Among these, preliminary treatment is designed to remove solids and large materials from raw wastewater. The primary treatment is sedimentation, which is the physical process that permits particles in wastewater effluent to deposit the suspension under the influence of gravity and thus become a sludge. The secondary step is anaerobic digestion, which is a biological treatment where the organic pollutants in the sludge are transformed by anaerobic microorganisms into gaseous products, such as methane. The final step is tertiary treatment, which is a chemical treatment that removes the organic load and effluent from the secondary treatment plants which contain nutrients, mainly phosphorus and nitrogen [132,133].

Many microcontaminants, especially pharmaceutical compounds with NSAIDs as the most commonly used class, cannot be fully removed by conventional wastewater treatment [23]. Thus, such contaminants may reach the aquatic environment as organic contaminants [135,136], which then pose a serious threat to food chains due to their tendency to bioaccumulate [137]. The main problem with discharging pharmaceuticals through conventional wastewater treatment is that drugs incorporate a wide spectrum of compounds with differences in their main properties. Such differences include (i) polarity, (ii) volatility, (iii) absorbability, (iv) adsorbability, (v) biodegradability, (vi) solubility and (vii) stability, which affect the behavior and fate of pharmaceuticals in wastewater treatment plants. Moreover, the concentration range for microcontaminants (from 10^−3^ to 10^−6^ mg L^−1^) is smaller than that for macrocontaminants (dissolved organic carbon, nitrogen compounds and phosphorus compounds) [20]. Other disadvantages of conventional processes are that (i) the toxicants cannot be fully removed, (ii) the equipment and monitoring systems used in these techniques are not cost-efficient, (iii) high reagent usage or energy are required and (iv) toxic sludge or other waste products are generated, which require a further adequate disposal process [138]. Thus, conventional wastewater techniques are inefficient and not well designed to remove micropollutants from contaminated wastewater [139].

Hence, besides the conventional approach, which may not be fully efficient due to the size and behavior of micropollutants, different approaches, such as adsorption, may be applied due to their capacities to remove soluble and insoluble organic pollutants [140]. Various methods are reported in the literature for the adsorption or removal of pharmaceuticals from water, including the use of different adsorbents, enzymatic treatment or advanced oxidation processes [141,142]. Advanced oxidation processes (AOPs) that belong to the most popular wastewater treatments are instead characterized by the formation of highly reactive and non-selective reactive oxygen species (ROS), which can mineralize organic compounds from contaminated matrices [143,144]. Depending on the ROS generation method used, AOPs can be divided into Fenton oxidation, photocatalytic oxidation, electrochemical oxidation, ozone oxidation, sonochemical oxidation and sulfate radical-based oxidation [145]. Since AOPs are considered to be the best method for wastewater remediation, they are also applied for pharmaceutical removal. Various AOP processes can remove pharmaceutical compounds from 40% to 100% depending on the technique used and experimental conditions [145]. Although AOPs have many advantages, such as no secondary pollution generation or high mineralization efficiency, they have also some limitations, including strict conditions required to make reactions efficient (pH, temperature, pressure, etc.) and high treatment costs [145]. Due to this limitation, more sophisticated methods are currently proposed, including the development of a variety of plasmonic materials to harvest light more efficiently for AOPs [146]. The possibility of pharmaceutical removal using plasmonic materials is not yet fully explored; however, the degradation of some drugs, including ibuprofen and levofloxacin, has been reported [146].

Enzymatic treatment involves biocatalytic conversion using living organisms or their enzymes, which are biologically made catalysts that facilitate biochemical reactions [142]. Studies of adsorption methods have demonstrated the potential of different adsorbents to reduce the concentration of NSAIDs, the most commonly used pharmaceuticals, in contaminated wastewater [147,148,149,150,151]. However, removal efficiency for diclofenac ranges from 14 to 69% [141], and for ibuprofen, ketoprofen and naproxen, it reaches only 40% [152]. Therefore, these processes may not be fully effective, and NSAIDs may be converted into other organic compounds; thus, the degradation products may not be environmentally friendly in the abovementioned approaches. 

Conventional wastewater treatment technologies are not able to remove emerging contaminants with high efficiency, rendering them ineffective in providing adequate clean water. As such, new methods are needed to obtain both considerable potable water savings through the reuse of wastewater and to investigate novel non-conventional clean-up techniques and water resources [130,153]. Seeking efficient methods of pharmaceutical removal and remediation, researchers have focused their attention on biological treatments of contaminated water. Such treatments include activated sludge in either aerobic or anaerobic conditions (anaerobic digestion), aerated lagoons, bioreactors and constructed wetlands [39]. Most of these techniques are based on the microbial (algal, fungal and bacterial) potential for adsorption, absorption and metabolization of pharmaceuticals, reflecting the ability of microorganisms to degrade pharmaceuticals by direct metabolic biodegradation or during cometabolic processes with other compounds [154]. Anaerobic digestion, the process of decomposition of organic matter using microbial organisms in oxygen-free conditions [155], involves the degradation and stabilization of organic materials and leads to the formation of biogas and microbial biomass [156]. Activated sludge is a method based on the biodegradation of the organic compounds in activated sludge tanks using aerobic or anaerobic microorganisms. The high toxicity of many contaminants prevents the application of this process in effluents with high pollutant concentrations, since they are recalcitrant and toxic to microorganisms [39]. Pharmaceuticals with high sorption coefficients segregate well with sludge and sediments [39]. However, this methodology can only be applied to effluents with high flow rates [157]. Closely related to activated sludge is another method called an aerated lagoon. This is used for the on-site treatment of landfill leachate, where treatment occurs via chemical and biological oxidation with surface aerators or by diffuse bubble aeration [158].

The use of bacteria and fungi for pharmaceutical bioremediation is often reported in the literature. In contrast, algal cultures are used mainly for the removal of nutrients and heavy metals and the potential of algae for the biodegradation of pharmaceuticals, and their mode of action for this application remains debated [39]. Nevertheless, it has been reported that some pharmaceuticals and their derivatives can be conjugated, sequestered and partially degraded by terrestrial and aquatic plants and algae [159,160]. Thus, algae-based remediation systems (phycoremediation) may represent promising methods for pharmaceutical removal from the environment.

## 4. Phycoremediation of Pharmaceuticals in Wastewater

The potential of algae to remove contaminants is called phycoremediation. This method has been considered for more than 50 years and has gained increasing attention in recent times, mainly due to rising population numbers and the increasing numbers of households and factories whose activities result in the release of large amounts of sewage [161]. Thus, phycoremediation is a promising economically and environmentally friendly biological treatment that uses macro- or micro-algae to remove or biotransform pollutants, such as heavy metals and organic contaminants, from wastewater, as well for CO_2_ sequestration [162,163]. Numerous examples of water contaminant removal using the phycormediation method with different algae species have been presented in the literature. The removal rate and removal time vary within the species used; however, the phycoremediation method may be efficient for the total removal (100%) of antibiotics, NSAIDs, β-blockers and other contaminants (Table 3).

Microalgae and macroalgae (seaweed) can be useful in phycoremediation. Macroalgae are considered to be efficient biosorbents for the removal of heavy metals and chemical dyes [164,165,166]. Seaweed use is, however, limited and can be challenging for scientists due to cultivation requirements (seawater, low tolerance of temperature and pH changes), the relatively slow growth rate of macroalgae and the lack of sustainable and sufficient natural sources of biomass [167,168,169]. Microalgae, as unicellular organisms, can grow much faster than macroalgae and are able to live under extreme environmental conditions, such as high salinity, nutrient stress and extreme temperatures. They are also relatively resistant to the presence of various pollutants (i.e., heavy metals, organic compounds or pharmaceuticals) [170,171]. Moreover, most microalgae can grow autotrophically, heterotrophically and mixotrophically [126]. Eukaryotic microalgae are also more genetically, enzymatically and chemically diverse than plants, fungi or animals, and they are characterized by a greater variety of primary and secondary metabolites, which may be relevant in the phycoremediation process [172]. 

Both macroalgae and microalgae are photosynthetic organisms that need abundant mineral nutrients, such as sodium, potassium, magnesium and calcium, as well as trace elements and carbon dioxide [173]. Thus, such algae are excellent for treating nutrient-rich municipal wastewater and sewage from the food industry (oil mills, wineries or breweries) [174,175]. Furthermore, the usefulness of algae in removing metals from the environment has been extensively discussed [176,177] and relates to the presence of internal and extracellular detoxification mechanisms in algal cells. One of the key elements of the reaction of microalgae with heavy metals is changing the expression of genes encoding selected proteins, and transporters responsible for processes such as the uptake, sequestration and detoxification of heavy metals [176,178]. Although the phycoremediation of nutrients and heavy metals is most often described in the literature, the use of this method to remove other pollutants, such as pesticides, dyes and pharmaceuticals, is reported in numerous papers. Despite anthropogenic contaminants varying in their chemical structures and physico-chemical properties, some of the mechanisms involved in phycoremediation appear to be unspecific and valid for substances of different types. 

**Table 3:** Examples of contaminants removed/potentially removed by selected algae species.

Type of Contamination	Substance	Algae Species	Removal Rate	Time	References
Antibiotic	Enrofloxacin (ENR)	*Platymonas subcordiformis* *Isochrysis galbana* *Scenedesmus obliquus* *Chlamydomonas mexicana* *Chlorella vulgaris* *Ourococcus multisporus* *Micractinium resseri*	75–85% *^,1^ 40–70% *^,1^ 23% 25% 26% 20% 26%	11 d 11 d 11 d 11 d 11 d	[179] [179] [180] [180] [180] [180] [180]
	Ciprofloxacin hydrochloride (CIP)	*Platymonas subcordiformis* *Isochrysis galbana* *Chlamydomonas mexicana*	65–85% *^,1^ 40–76% *^,1^ 13–56% ^2^	11 d	[179] [179] [180]
	7-amino cephalosporanic acid (7-ACA)	*Chlorella* sp. Cha-01 *Chlamydomonas* sp. *Tai-03* *Mychonastes* sp. *YL-02*	>70% 70% * 65% *	24 h 24 h 24 h	[181] [181] [181]
	Cefradine (CFD)	*Chlamydomonas reinhardtii* *Chlorella pyrenoidosa*	5–14% 41%	8 h 24 h	[182] [183]
	Amoxicillin	*Chlorella pyrenoidosa*	91%	6 h	[183]
	Clarithromycine	*A mixed population of wild freshwater green algal species (Dictyosphaerium)*	90%	7 d	[184]
NSAID	Ibuprofen	*Chlorella pyrenoidosa* *Chlorella sorokiniana* *Nannochloropsis* sp. *Scenedesmus obliquus*	29–31% 100% * 51–100% -	42 d 31 d 10 d -	[185] [186] [187] [188]
	Diclofenac	*Chlorella sorokiniana* *Chlorella sorokiniana* *Chlorella vulgaris* *Picocystis* sp. *Graesiella* sp. *Scenedesmus obliquus*	40–60% 30% 22% 73%, 43% and 25% (25, 50 and 100 mg L^−1^) 52%, 28% and 24% (25, 50 and 100 mg L^−1^) 79%	31 d 9 d 9 d 9 d	[186] [189] [189] [189] [190] [189]
	Naproxen	*Scenedesmus quadricauda*	59%, 73%, 2% (1, 10 and 100 mg L^−1^	30 d	[109]
	Paracetamol	*Chlorella sorokiniana* *Chlorella sorokiniana* *Nannochloropsis* sp	100% * >67% from 50.5 to 44.4 μg mL^−1^	31 d 8–9 d 24 h	[186] [191] [187]
β-blocker	Atenolol	*A mixed population of wild freshwater green algal species (Dictyosphaerium)*	99%	7 d	[184]
	Bisoprolol	*A mixed population of wild freshwater green algal species (Dictyosphaerium)*	97%	7 d	[184]
	Metoprolol	*A mixed population of wild freshwater green algal species (Dictyosphaerium)* *Chlorella sorokiniana*	99% 100% *	7 d 31 d	[184] [186]
Other drug	Alfuzosin Atracurium Bupropion Citalopram Clonazepam Dicycloverin Diltiazem Diphenhydramin Hydroxyzine Memantin Miconazole Pizotifen Terbutalin	*A mixed population of wild freshwater green algal species (Dictyosphaerium)*	64% 97% 93% 98% 88% 71% 94% 89% 87% 81% 65% 80% 98%	7 d 7 d 7 d 7 d 7 d 7 d 7 d 7 d 7 d 7 d 7 d 7 d 7 d	[184]
	Carbamazepine	*Chlorella sorokiniana*	30%	7 d	[186]
	Trimethoprim	*Chlorella sorokiniana*	60%	7 d	[186]
	Salicylic acid	*Chlorella sorokiniana*	>73%	8-9 d	[191]

* approximately; ^1^ depending on the initial concentration of the pharmaceutical; ^2^ depending on the concentration of sodium acetate.

### 4.1. Mechanisms of Phycoremediation

**Figure 2:**
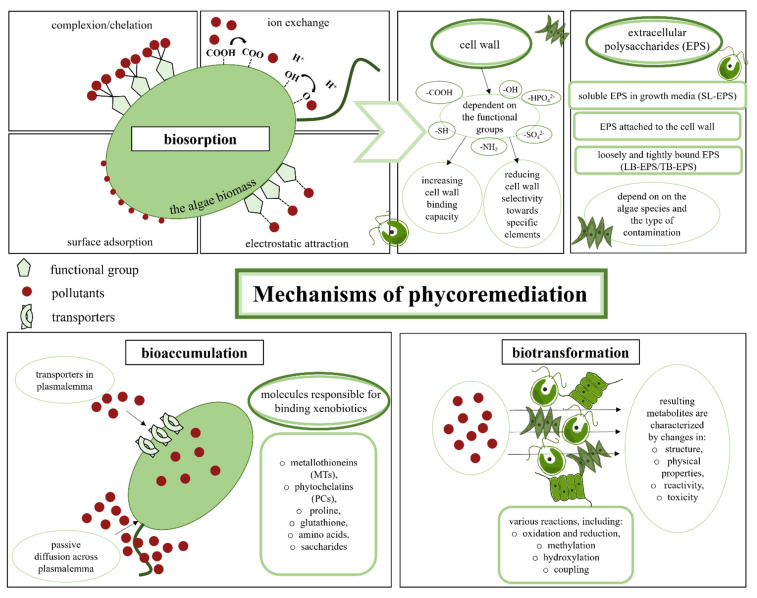
Mechanisms of phycoremdiation.

Phycoremediation can be based on both extracellular and intracellular processes, such as biosorption, bioaccumulation, sequestration and biotransformation/biodegradation (Figure 2). Additionally, algae can simultaneously use several mechanisms that complement each other and increase the effectiveness of removing pharmaceuticals and other toxic substances from the environment [186,192]. Biosorption and bioaccumulation processes are amongst the most intensively investigated remediation techniques; however, these two terms are sometimes confused. Biosorption, usually defined as the passive binding of toxicants by dead (inactive) biomass or by materials derived from biomass, consists of a set of metabolism-independent (physico-chemical) processes primarily connected with cell walls. Conversely, bioaccumulation is the process of the uptake and intracellular accumulation of toxicants by living cells [193,194].

Heavy metals, dyes, drugs and other chemical contaminants can be adsorbed by the cell wall or bound by extracellular polysaccharides (EPS). Scientists have focused their attention on EPS, the synthesis of which is closely related to the response of algae to stress. The composition and properties of EPS may vary depending on the algae species and the type of contamination. Thus, EPS has been divided into three groups: (i) soluble EPS in growth media (SL-EPS), (ii) attached EPS to the cell wall and (iii) loosely and tightly bound EPS (LB-EPS/TB-EPS), which provides additional protection to the wall [195,196,197]. The adsorption capacity of the cell wall itself depends mainly on the functional groups of polysaccharides and proteins that build it, including -COOH, -OH, -HPO_4_^2−^, SO_4_^2−^, -NH_2_ and -SH groups. These groups can act in two ways: by increasing the binding capacity of the cell wall, and by reducing its selectivity towards specific elements [198,199]. Among the mechanisms of biosorption, several chemical and physical processes are distinguished (Figure 2). The first of these, chelation/complexion, consists of the incorporation of mineral ions into a complex structure by an organic molecule called the chelating agent. Generally, sulfur, nitrogen and/or oxygen are electron-donor atoms on the chelating molecule [200]. Furthermore, the formation of hydrogen bonds between cell wall components and xenobiotic molecules, including pharmaceuticals, must be considered. For example, hydrogen bond formation is the main method of sulfamethoxazole and sulfacetamide biosorption by marine algae [201]. Another mechanism of biosorption is ion exchange, occurring when pollutants, usually heavy metal ions, displace other metals from the functional group and adsorb onto the algae wall. The main task of the physical forces, mainly van der Waals and electrostatic interactions, is to direct the physical mechanism of adsorption of the pollutant bond with the cell surface [202,203]. 

The literature indicates that the bioadsorption capacities of microalgae towards pharmaceuticals vary significantly depending on the strain and pharmaceutical studied, ranging between 0 and 16.7% when diclofenac, ibuprofen, paracetamol, metoprolol, trimethoprim, carbamazepine, estrone, b-estradiol, progesterone, norgestrel and ethinylestradiol are considered [180]. The bioadsorption capacity of *Chlorella* sp., *Chlamydomonas* sp. and *Mychonastes* sp. towards 7-amino cephalosporanic acid ranges from 4.74 to 2.95 mg/g, whereas *Scenedesmus quadricauda* and *Tetraselmis suecica* can adsorb 295.34 and 56.25 mg/g of tetracycline, respectively [204]. Moreover, authors have indicated that microalgae efficiency in antibiotic bioadsorption ranges from 7.3% for sulfamethazine removed by *Scenedesmus obliquus* to 100% for metronidazole removed by *Chlorella vulgaris.*

Overall, the efficiency of biosorption in contaminant removal is comparable to chemical methods of remediation; however, the advantage of the former is related to the lower cost of biosorbents, their wide availability, the large number of binding sites they possess and their high adsorption capacity. Moreover, algae biomass can also be used for other processes, such as the production of biofuels and biochar [205,206,207].

Biosorption, a fast physico-chemical process, is also the first step of contaminant removal when living algal cells are used for phycoremediation. However, part of the toxicant enters the cell interior over time with exposure. This relatively slow transport occurs either via transporters in plasmalemma (e.g., metal ions) or via passive diffusion across the membrane (e.g., lipophilic organic molecules), leading to the bioaccumulation of chemicals. Most pharmaceuticals, as relatively high molecular mass and lipophilic molecules, enter the cell interior through passive cell membrane diffusion, as has been demonstrated for triclosan and triclocarban bioaccumulated by *Cladophora* sp.; carbamazepine concentrated by *Pseudokirchneriella subcapitata, Chlamydomonas mexicana* and *Scenedesmus obliquus*; and florfenicol accumulated by *Chlorella* sp. [204,208]. 

Once toxicants enter the cell interior, they can be sequestrated by cell compartments either through physical compartmentation or binding to specific macromolecules. The group of molecules responsible for binding numerous xenobiotics encompasses metallothioneins (MTs), phytochelatins (PCs), proline, glutathione, some amino acids and saccharides [177,209]. Most of the literature concerning the sequestration of various contaminants into algal cells primarily describes heavy metal and nutrient remediation [210], with only limited papers referring to other types of contaminants. The sequestration of halogenated organic compounds, such as trichloroethylene and tetrachloroethylene by *Spirogyra* spp., *Nitella* spp. and photoautotrophic cyanobacteria (*Oscillatoria* spp., *Nostoc* spp. and *Anabaena* spp.) was described [211]. The uptake and sequestration of herbicide, pesticide and petroleum compounds by microalgae have also been reported [212]. Regarding pharmaceuticals, results have shown that, in plant and algal cells, drug conjugates with glucuronic acid, sulfate, amino acids, tyrosine and glutathione can be formed [192,213]; this is a transient state that precedes the biotransformation step. Biotransformation is the process by which xenobiotics are metabolized, and the resulting metabolites are characterized by a change in structure, physical properties, reactivity and, above all, toxicity. Biotransformation in algal cells can occur via many different pathways, depending on the characteristics of the xenobiotics [213,214,215]. Pathways of xenobiotic biotransformation in algal cells have been reported primarily for heavy metals. Species such as *Chlorella vulgaris, Symbiodinium minutum, Chlorella fusca* and *Galdieria sulphuraria* have enormous potential for the detoxification of chromium or mercury [216,217,218]. Investigations of the mechanism of arsenic biotransformation in algal cells have revealed that this element can be oxidized, reduced and then methylated. As a result of further reactions, it was transformed into arsenosugars, among other products. Arsenic–GSH complexes can also be formed [214]. Although the metabolic pathways for the transformation of organic xenobiotics by algae are less-known, a few papers report that some dyes, pesticides and pharmaceuticals are metabolized by green algae and cyanobacteria [216,217,219]. Thus, 17α-ethynylestradiol, 17b-ethynylestradiol, estriol and estrone can be transformed by the green algae *Desmodesmus subspicatus* and *Scenedesmus dimorphus*. Interestingly, 17a-estradiol and 17b-estradiol are initially transformed into estrone, which is metabolized to form estriol and then further degraded into unidentified products. Moreover, triclosan can be transformed by *Microcystis aeruginosa* with methylation to methyl-triclosan as a major biotransformation pathway [215]. In addition, biotransformation is reported to be the major mechanism for the elimination of progesterone and norgestrel by *Scenedesmus obliquus* and *Chlorella pyrenoidosa*. Hydroxylation, reduction and oxidation reactions are involved in the pathways used to convert these hormones [220]. In one of the most extensive research studies, Stravas et al. [213] have demonstrated that *Microcystis aeruginosa*, *Synechococcus* sp. and *Chlamydomonas reinhardtii* are able to transform eight xenobiotics (strobilurin, mefenamic acid, atenolol, metoprolol, sulfamethoxazole, bezafibrate, ranitidine and verapamil) via different enzymatic reactions such as hydrolysis, CYP450 oxidation reactions, methylation and conjugation with glutamate and pterin.

### 4.2. Selected Factors Affecting Phycoremediation Efficiency

**Figure 3:**
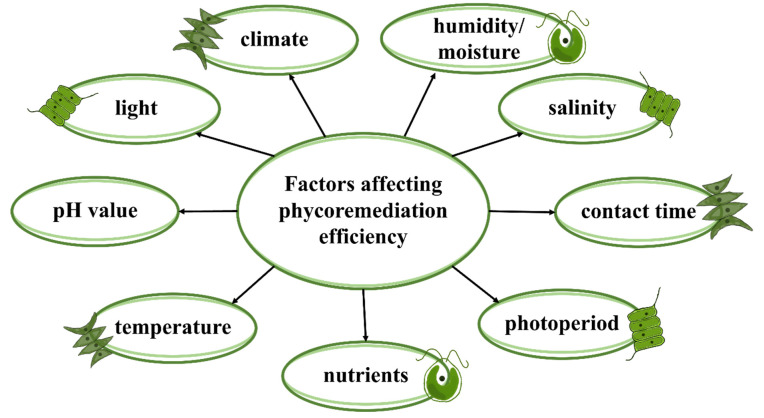
Factors affecting phycoremediation efficiency.

The effectiveness of phycoremediation is influenced by many factors, including the algae species used in the process, characteristics of the toxicant being removed, temperature, pH, availability of light, oxygen, nutrients, humidity/moisture, climate and salinity (Figure 3). Thus, each of the parameters needing to be applied in a particular phytoremediation system requires optimizations of intensity and value; this is one of the probable reasons why the literature still lacks comprehensive data on the influence of individual factors on the ability of selected strains to remediate pharmaceutical contaminants. Therefore, different factors such as light, pH value and temperature should be considered when planning algae systems to remove contaminants.

#### 4.2.1. Light

Light is a crucial factor influencing the growth and productivity of algae because these organisms are photoautotrophs that use light energy to perform the process of photosynthesis and produce metabolically useful energy in the form of ATP and NADPH[+H^+^]. All processes occur in photosystems in which chlorophylls and carotenoids are responsible for light absorption [219,221]. Both the wavelength and the intensity of light are relevant. The photosynthetically active radiation (PAR) used by algae is between 400 and 700 nm; however, green algae (Chlorophyta and Charophyta), red algae (Rhodophyta), brown algae (Phaeophyta) and cyanobacteria (Cyanophyta) vary in their preferences with regard to both the quality and quantity of light. Nevertheless, the greatest efficiency of phycoremediation is observed in the blue and red regions of the light spectrum. For example, in studies on *Scenedesmus* sp., high efficiency of nitrogen and phosphorus removal, as well as increased algae growth rate, were obtained by mixing red and blue light [222]. Similar results were obtained in studies focused on the upgrade of biogas by nutrient removal from biogas fluid by *Chlorella* sp. [223]. Such results are not surprising, since light, especially in the wavelength range corresponding to blue and red, is a key environmental factor for photosynthetic organisms, not only as an energy source but also as a signal to modulate various developmental processes (photomorphogenesis). Both blue and red light are responsible for the activation of many biochemical pathways and are factors that modulate gene transcription. Therefore, exploiting light and combining it with biocatalysis can greatly improve “green chemistry” and open new opportunities for biosynthetic reactivities [224]. For instance, blue-light photoreceptors of the Light, Oxygen and Voltage (LOV) family are currently being investigated in the context of their usefulness in enzyme bioengineering to design light-controlled biocatalysts [225].

The other aspect of light influence on phycoremediation is its intensity. In special systems that enable phycoremediation, such as high-rate algae ponds or bioreactors, low-efficiency light use by algae has been identified as a problem. This is due to the depth of the reservoirs, the high density of algae and poor mixing results in some of the algae population, such as algae grown next to the bottom of the pond, thus suffering from light deficiency [226]. Therefore, it is important that the conditions of culture are carefully selected [227]. Many cultivating systems use an artificial light source to improve photosynthesis and phycoremediation efficiency. This often generates higher costs and is why many studies focus on choosing the most appropriate and economical lighting source [223,228].

Another factor influencing phycoremediation is photoperiod. This is not directly related to the mechanisms of phycoremediation; however, it affects the production of biomass and lipids, the composition of cells, and their growth rate [229]. It has been shown that the extension of the dark period in the photoperiod increases the efficiency of carbon removal by *Coelastrum* sp. from municipal wastewater. However, for nitrogen and phosphorus, the efficiency of this process decreases [228].

#### 4.2.2. pH Value

One of the factors that is difficult to maintain at a constant level, especially in open remediation systems, is pH. Significant changes in pH are often observed for photoautotrophic cultures, including aerated ponds or sequential batch reactors used for tertiary treatment of wastewater [230]. Alkalization of the growth media (above pH 9.0) can reduce the effectiveness of wastewater purification by inhibiting the growth of toxicant-degrading bacteria and algae [231]. Furthermore, in the process of phycoremediation, the pH value affects the efficiency of the sorption of chemicals on the cell surface, where a large number of carboxyl groups, protonated at a low pH, are present. As the pH increases, the carboxyl group and other negatively charged groups deprotonate, increasing the remediation efficiency as a result of the electrostatic attraction of positively charged particles [232,233]. In the pathways of biotransformation for pollutants, enzymes are of great importance. Although live algae cells can maintain internal homeostasis at a wide range of external pH levels, thus protecting their enzymes from inactivation, in in vitro systems, enzyme bioactivity is strictly affected by pH. In studies on the biotransformation of carbamazepine by the laccase-mediator system, it was demonstrated that pH and temperature affect its removal. This pharmaceutical has been almost completely degraded by a system with pH in the range of 5.5 to 6.0 [234]. Zhang and Geißen [235] investigated in vitro degradation of carbamazepine and diclofenac by crude lignin peroxidase. These authors observed that diclofenac was degraded at a pH of 3.0 to 4.5. Therefore, determining the optimal pH value for the phycoremediation process requires knowledge of the mechanisms of drug degradation and the pathways of drug biotransformation, including the enzymes responsible for this course. 

#### 4.2.3. Temperature

Another important factor influencing phycoremediation is temperature, especially when live algae cells are used. The influence of temperature can be particularly observed in open systems, where algae are exposed to daily and seasonally dependent temperature fluctuations. Individual species of algae require different temperature ranges for growth, usually from 15 to 30 °C [236,237]. At low temperatures, the metabolism of algae decreases; therefore, the effectiveness of remediation is reduced. Conversely, temperatures that are too high adversely affect the growth of algae and can damage cells [238,239]. In addition to the key role of temperature in the growth of algae biomass, the temperature is also important for biotransformation in systems where enzymes are used *in vitro*. In addition to the appropriate pH, enzymes need the appropriate temperature to work efficiently. For example, temperature increases the removal of carbamazepine by laccase, and the efficiency of this process at 35 °C is 100%. Lowering and increasing the temperature by 10 °C results in a decrease in productivity by about 30% [234]. Cerveny et al. [240] showed the importance of temperature in the biotransformation of temazepam in fish. The effects obtained at 20 °C were better than at 10 °C. Furthermore, microalgae, *Acutodesmos acuminatus,* used as a biosorbent for europium, also required a suitable temperature for the process, and the maximum adsorption capacity was achieved at 40 °C. It has been suggested that proteins are involved in the biosorption process because, at 50 °C, the efficiency of the process was shown to decrease, and the algae were already dead [241].

#### 4.2.4. Other Factors

Nutrients, primarily associated with the growth of algae, are important factors influencing the efficiency of phycoremediation. One of them, carbon, is a biogenic element necessary for the development of any living organism; therefore, considering the possibility of the mixotrophic growth of algae, both inorganic and organic carbon forms play important roles in algae cultivation. Thus, wastewater is an ideal source of carbon for algae growth [231,242]. The influence of different types of carbon on the growth and life processes of algae has been extensively described by Zhan et al. [243] and Kaloudas et al. [231]. Other important elements influencing the accumulation of biomass are nitrogen and phosphorus. The content of these compounds should be determined earlier in the drain, as it has been confirmed that their ratio to each other has an effect on the growth of the microalgae and their ability to bioremediate various substances [244,245]. 

Air humidity/moisture and climate are other factors that influence phycoremediation efficiency in algae-based systems. These factors regulate the process of water evaporation from the open ponds or matrix used for algae immobilization, thus influencing water availability for algal cells. However, it is difficult to predict evaporation intensity and to maintain adequate water balance, especially in open ponds [246,247]. Increased phycoremediation efficiency can also be obtained by modulating the time of contact of algae with contaminated water [221,247]. Some scientists have also suggested salinity as a factor relevant to algae remediation systems influencing algae growth [248]. However, there is a lack of literature describing the direct impact of salinity on the removal of pharmaceuticals from sewage.

## 5. Algae-Based Remediation Systems

The potential benefits of using higher plant cultures in phytoremediation-focused research have been widely studied and are convincing [10,249,250]. However, this methodology does have disadvantages and limitations. The higher plants cultures are strictly dependent on season, and not all species are fast-growing and able to produce a large amount of biomass in a relatively short time. Thus, the time required for the removal of the contaminants from matrices can be exceeded. Moreover, cell heterogeneity of higher plant-based systems may cross-influence between different plant tissues or organs, and cultured cells may impact the reproducibility of results. Most of the abovementioned issues with higher plant cultures can be eliminated by using microalgae-based experimental culture systems, because these cultures, once established, (i) are available independently of the season, (ii) can be continuously propagated, (iii) can be maintained under strictly controlled conditions, (iv) allow a large amount of biomass to be produced in a relatively short time, (v) are fast-growing, thus the time required to carry out the experiments may be significantly reduced, (vi) help to improve the reproducibility of the results due to cell homogeneity and eliminating possible barriers found in higher plants such as root epidermis and endodermis, xylem translocation, etc., and (vii) do not have the cross-influence between different plant tissues or organs. In contrast with strictly heterotrophic microorganisms, reductions in nutrient concentration only slightly limit the growth of algae [150]. Moreover, many microalgae species can adapt to extreme environmental conditions, explained by genetic changes caused by spontaneous mutations or physiological adaptations, which improve their biodegradation capacity [126,251]. Cultures of unicellular algae are thus a good tool in phycoremediation research to investigate the biochemical responses of plant cells to environmental contaminants. Selected microalgae strains, highly adaptable and resistant to chemically-induced stress, can therefore be used to efficiently remove toxicants from wastewater [252].

In algae-based systems, open ponds are commonly used for macroalgae and microalgae cultivation due to their low operational, capital and investment costs. Facultative, high-rate and maturation algal ponds, which differ primarily in depth and the origin of their influent, are widely used open systems for wastewater treatment [126]. Among them, large shallow raceways and circular and unstirred open ponds are the most commonly applied outdoor approaches for large-scale algal cultivation [253]. Open systems usually function under long hydraulic retention times to consume CO_2_ during the day and provide O_2_ for aerobic biodegradation. This is because sunlight intensity influences photosynthetic activity, leading to pH and dissolved oxygen variations [126,254]. Due to differences in the major limitations of these systems, low productivity and risk of contamination are usually reported (Table 4) [255,256].

Photobioreactors, described as illuminated and enclosed vessels intended for controlled biomass production [257], are examples of closed culture systems. Closed systems based on photobioreactors, even if they are expensive to install and maintain, allow greater control of the process [126]. Based on the mode of liquid flow, photobioreactors can be divided into stirred type, bubble column and airlift reactors. Depending on the illuminated surface, photobioreactors may be categorized as tubular [258], flat plate [259] or column [260]. Sustainable closed-system photobioreactors thus occur in numerous design configurations based on different systems, such as flat-panel, horizontal tubular, stirred tank, helical and vertical-column (with two categories: bubble column and airlift column) (Table 4). The photobioreactors for suitable algae cultivation and their mechanisms were extensively described by Singh et al. [257], Gupta et al. [261], Ugwu et al. [255], Molina et al. [258] and Slegers et al. [259]. Closed systems are most suitable for axenic algae strains and must be carefully adjusted for each individual strain according to its growth and physiological characteristics. These systems reduce contamination risk, avoid water losses by evaporation and prevent losses of CO_2_ to the atmosphere [262]. Photobioreactors require in-depth knowledge of different factors, such as scalability, mass transfer, light distribution, shear stress and the biology of algae cells. The photobioreactors described in the literature generally do not fulfil all of the aforementioned requirements. Thus, further efforts are required to combine different types of bioreactors to develop suitable hybrid bioreactors for mass algal cultures [257].

**Table 4:** The major characteristics of algae cultivation systems, based on [261].

**Cultivation System**	**Mixing**	**Temperature**	**Gas** **Exchange**	**Limitations**	**Advantages**	**References**
Open systems						
Open ponds	Paddle wheel	None	Limited, through surface aeration	Less control over culturing conditions; temperature fluctuations; poor light utilization by the cells; inefficient stirring; diffusion of carbon dioxide to the atmosphere; lower biomass productivity; risk of contamination; large land space requirement	Simple design; cost-efficient; low investment costs; not difficult to maintain	[256]
Closed systems						
Vertical column photobioreactors(bubble column photobioreactors and airlift columns)	Airlift or bubble	-	Open gas exchange at head space	Expensive construction materials; limited scale-up opportunities due to design constraints and inhomogeneous distribution of light inside the culture; productivity negatively affected by light-deprived zones; limited surface area for illumination; shading effect issues; photosynthetic efficiency depends on gas flow rate	Efficient mixing; high volumetric mass transfer rates; relatively homogenous culture environment; low photoinhibition; controllable growth conditions; lack of moving parts; no internal structures	[260,263]
Stirred-tank photobioreactors	Mechanical agitator	Heat exchanger	Injection through sparger	Not cost-efficient; mechanical agitation requires extra energy; low surface-to-volume ratio; low harvesting efficiency; heating issues due to agitation	Appropriate light dispersion; appropriate heat and mass transfer; simple design; moderate biomass; low contamination issues; productive	[257,263]
Flat-panel photobioreactors	Airlift or bubble from bottoms or side or rotating mechanically through motor	Heat exchangecoils	Open gas exchange at head space	Requires many components; short light penetration depth; frequent fouling and clean up issues; not scalable; poor temperature regulation	Cost-efficient; low space requirement; high surface-to-volume ratio; high photosynthetic efficiency; low oxygen build-up	[255,257,263]
Horizontal tubular photobioreactors	Recirculation via pumps	Water spraying; shading; overlapping	Injection into feed	Large space requirement; high energy consumption; susceptible to photo inhibition; dissolved oxygen buildup; fouling due to algal growth; poor temperature regulation	Cost-efficient; harnessing sun light efficiently; suitable for outdoor cultivation; high surface-to-volume ratio; low hydrodynamic stress; good biomass productivity; low mutual shading effect	[257,263]
Helical-type photobioreactors	Centrifugal pump (injection from bottom)	Heat exchanger	-	Limited commercial use associated with shear stress; fouling on the inside of the reactor	High photosynthetic efficiency through the light dilution effect and light absorbing capacity; high CO_2_ transfer; balance between energy input and photosynthetic efficiency; low energy requirement; low mechanical stress to cells	[257,264]

**Figure 4:**
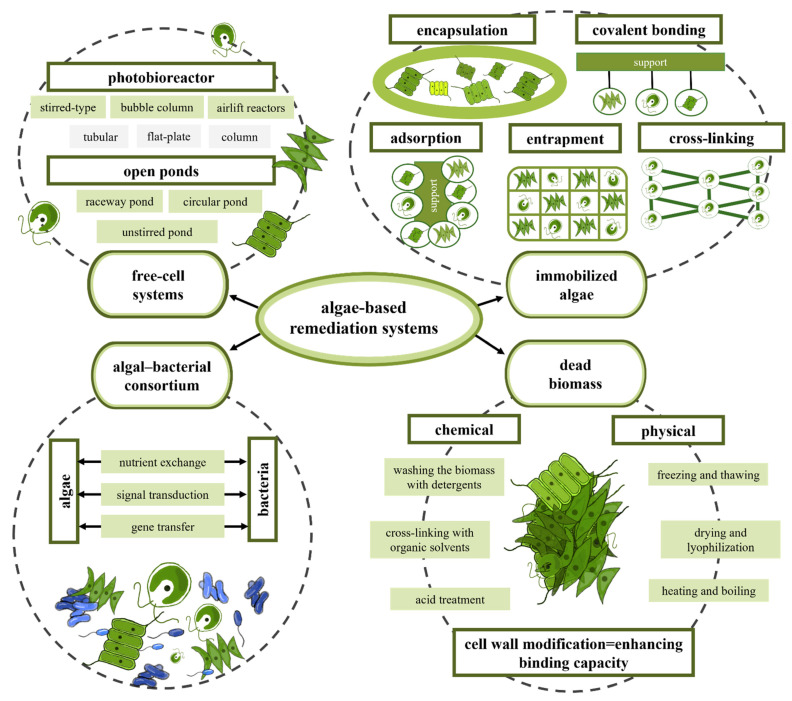
Algal-based remediation systems based on [253,265,266,267,268].

There are many variants to the different open and closed algal-based systems for wastewater treatment described above, including variants where free cells, immobilized algae, algal-bacterial symbiotic consortia or dead biomass as a biosorbent are used (Figure 4).

### 5.1. Free Cell Cultures and Immobilized Algae

Most algal cultivation systems, including open and closed systems, and research related to the phycoremediation of wastewater are based on free algal cells [126,179,184,185,186,187,190,269,270,271]. The most widespread system utilizes shallow and high-oxygenated open ponds with free cells that favor the intensive growth of microalgae. However, a major disadvantage of this approach is associated with the high cost of harvesting the microalgae. Research efforts have thus aimed to use non-suspended, immobilized algae to avoid the harvesting issue, as well as to provide other benefits such as improvements in metabolism, function and behavior; a reduction in competition for nutrients with other microbial species; and an increase in cell retention time within bioreactors [272]. 

Thus, in non-suspended immobilized algae cultures, immobilized cells are blocked and cannot migrate independently to parts of the aqueous phase of the system by organic or inorganic carriers [273]. Different mechanisms of immobilization of microbial cells include covalent coupling, adsorption, encapsulation into a polymer gel, cross-linking of microorganisms and entrapment in a matrix (Figure 4). These mechanisms have been extensively reviewed by Bouabidi et al. [265]. Different carriers are used for the immobilization of viable microbial cells, and the selection of carriers depends on factors such as cost-effectivity, good mechanical strength, light weight, flexibility, nontoxicity and non-biodegradability under test conditions. Organic and inorganic carrier materials are mostly applied in the immobilization of microorganisms. Organic polymer carriers can be divided into natural and synthetic polymers [265]. Alginate, carrageenan, agarose, chitosan, agar and collagen are frequently adapted natural organic carrier materials [274]. Synthetic polymers such as polyvinyl alcohol, glycol, polyacrylamide, polycarbonyl sulphonate, polyethylene and synthetic plastics have been adapted as carrier materials. Inorganic carrier materials such as ceramics, clay, anthracite, porous glass, activated charcoal and zeolite are the most frequently used inorganic carriers due to their cost-efficiency, thermostability performance and resistance capacity to microbial degradation [265]. 

The key purposes of immobilizing algal cells are to retain living cells with limited mobility during their functioning within a matrix and to keep cells metabolically active for as long as possible [275]. Microalgal cells immobilized by different carriers are mostly utilized for phycoremdiation of different heavy metals, nitrogen and phosphorus from contaminated wastewater. This is because microalgae serve as a good biosorbent, providing a high sorption capacity for metals and nutrients. Moreover, the commonly used carriers used for immobilization are organic carriers such as carrageenan and alginate in different forms as gels and beads (Table 5) [265,276]. However, further investigation regarding the use of immobilized algal cells for the removal of pharmaceuticals is needed. 

### 5.2. Algal–Bacterial Consortiums 

In the algal–bacterial consortium, microalgae provide O_2_ for aerobic bacteria to degrade organic matter and to consume CO_2_ produced by bacterial respiration. Moreover, microalgae and bacteria may form flocs that settle more easily than a single microalgae culture (Table 6). Different algal–bacterial interactions, namely, nutrient exchange, signal transduction and gene transfer mechanisms, allow them to coexist in symbiotic consortia (Figure 4), and these have been extensively described by Jiang et al. [266]. The effectiveness of algal–bacterial consortiums is related to abiotic factors such as light intensity, pH, nutrient load, temperature, inoculum dose and CO_2_, and biotic factors such as the pathogens present in wastewater [255,288,289]. Choosing the appropriate algal and bacteria strains for different types of wastewaters demands in-depth knowledge of the mechanisms of the interactions in the consortia [290,291]. 

Even if the removal efficiency with algal–bacterial consortia is high for pharmaceuticals, nutrients or metals (up to 100%, 100% and 70%, respectively; Table 6), the main difficulties in consortia applications are (i) the variability of wastewater, (ii) the large area required, (iii) low hydraulic retention and (iv) that effluent quality deterioration limits their scale-up application [292]. However, the major advantages of using algal–bacterial symbiotic systems are the reduced requirement for aeration and more efficient nutrient removal. Therefore, these systems are an economically suitable alternative to conventional aerobic treatments for the clean-up of wastewater; thus, the use of consortium systems in the treatment of domestic and industrial wastewater has gained attention in recent years [293]. Algal–bacterial consortia have been exploited for disinfection and the removal of nutrients, pharmaceuticals and heavy metals from wastewater (Table 6). Multiple studies have demonstrated that more efficient and advanced nutrient and contaminant removal from wastewater is achieved through a combination of algal and bacterial systems, rather than through using single algal or bacterial systems (Table 6) [248,294].

**Table 6:** Removal efficiencies of contaminants with selected algal–bacterial consortia.

Consortium	Class of Compounds	Compound	Cultivation System	Removal Rate	Contaminated Matrix	References
Pharmaceuticals						
*Chlorella vulgaris* with heterotrophs	Antibiotics	Tetracycline	High-rate algal ponds	69%	Urban wastewater	[226]
*Chlorella* sp., *Pseudomonas aeuroginosa,* *Pseudominas* sp. with *Stenotrophomonas*	A/A A/A NSAID NSAID	Paracetamol P-aminophenol Ketoprofen Salycilic acid	Stirred-tank packed-bed reactor	100% 100% 98% 95%	Urban wastewater	[295]
*Artemia* sp., *Spirulina* sp. with bacterial consortium	NSAID	Ketoprofen		5 mM	Wastewater effluents	[296]
*Algal–bacterial consortium* from high-rate algal ponds	NSAID NSAID NSAID	Ibuprofen Naproxen Salicylic acid Triclosan Propylparaben	Photobioreactor operating at a hydraulic retention time	94% 52% 98% 100% 100%	Urban wastewater	[292]
Nutrients						
*Chlorella vulgaris* with *Bacillus licheniformis* and *Microcystis aeruginosa* with *Bacillus licheniformis*	-	TDN TDP COD	Reactor (conical flask)	89% 80% 87%	Synthetic wastewater	[288]
*Scenedesmus quadricauda* with bacteria from nitrogen-enriched activated sludge	-	NH_4_^+^		100%	Synthetic wastewater	[289]
*Chlorella vulgaris* with bacteria	-	P DOC NH_4_^+^	Tabular photobioreactor	98% 26% 97%	Municipal wastewater	[297]
*Scenedesmus* sp. with bacteria	-	COD TN TP		92% 95% 98%	Municipal wastewater	[298]
*Chlamydomonas* and *Euglena with cyanobacteria,* *Microcystis aeruginosa*		COD TN NH_4_^+^ TP BOD_5_	Waste stabilization pond	78% 87% 99% 97% 89%	Domestic wastewater	[299]
*Chlorella vulgaris* with bacteria	-	N TP COD		100% 100% 90–95%	Synthetically made municipal wastewater	[300]
Metals						
*Ulothrix* sp. with bacteria consortium	-	Cu Ni Mn Zn	Laboratory-scale photo-rotating biological contactor	50% 50% 40–45% 35%	Drainage wastewater	[11]
*Chlorella* sp., *Chlorella* sp. and *Scenedesmus obliquus* with *Rhodococcus* sp. and *Kibdelosporangium*	-	Cu Ni Mn	-	62% 62% 70%	Industrial wastewater	[12]
*Chlorella sorokiniana* with *Ralstonia basilensis*	-	Cu	-	8.5 mg/g	Synthetic wastewater	[301]

A/A: analgesics and antipyretics; BOD: biochemical oxygen demand; TP: total phosphorus; TN: total nitrogen; DOC: dissolved organic carbon; COD: chemical oxygen demands.

### 5.3. Dead Biomass as a Biosorbent

Biosorption is a process that uses the dead biomass of algae as a biosorbent to sequester heavy metals or organics from aqueous solutions [194]. Algae are of interest in the development of new biosorbent materials due to their unlimited quantities in water bodies, the positioning of functional groups on their surface (cell wall) that serve as binding sites for metals and their high sorption capacity [4]. Wang and Chen [268] extensively described and listed the high biosorption capacity of different algae strains for different heavy metals. The higher sorption capacity of algae is due to their cell wall being composed of a fiber-like structure and an amorphous embedding matrix of various polysaccharides [302]. Moreover, the biosorption process depends on the cell surface; thus, modification of the algal cell wall can greatly alter the binding of ions. As such, several methods have been employed for cell wall modification to enhance the metal binding capacity of biomass. The chemical treatments used for biomass modification include washing the biomass with detergents, cross-linking with organic solvents and acid treatment. Physical methods include freezing and thawing, heating and boiling, or drying and lyophilization [268]. Biosorption of metals may also be enhanced by heat, chemical sterilization or crushing [303].

Many studies have demonstrated that dead algal biomass may be even more effective than living algae in sequestering heavy metals [304,305]. The major advantages of using dead biomass in biosorption include (i) low cost, (ii) high efficiency of heavy metal removal from diluted solutions, (iii) minimizing the formation of chemical and/or biological sludges, (iv) no nutrient requirements for microorganism growth and the absence of toxicity limitations and (v) the possibility of metal recovery and regeneration of the biosorbent [162,268,306]. Moreover, biosorption can produce high-quality clean effluents, and due to the reversible adsorption process, the biosorbents used can be renewed through desorption in some cases [307]. The use of inactivated biomass also has disadvantages, such as having no scope for biosorption improvement through mutant isolation, the impossibility of using dead cells if biological alteration in valency of a metal is pursued and the inability for the degradation of organometallic species [308]. 

In the past years, biodegradable polymeric (nano)adsorbents based on algal poly-mers, e.g., alginate and cellulose, have also been developed. They are successfully used to adsorb various ubiquitous organic pollutants, such as heavy metals, phenolic compounds, aromatic or polyaromatic hydrocarbons, alkanes and their derivatives [309,310]; however, they are rarely used for pharmaceutical removal. The limited research about the use of cellulose in the form of nanocellulose crystals (CNCs) for pharmaceutical removal has shown that chemically modified CNCs have the capacity to adsorb drugs such as doxorubicin hydrochloride, tetracycline hydrochloride, docetaxel, paclitaxel, procaine hydrochloride and salbutamol [311].

Considering all of the abovementioned issues, biosorption offers advantages over conventional processes. Dead biomass and (nano)adsorbents are mostly used as a biosorbent to sequester heavy metals and phenolic compounds; thus, further studies regarding algae and algae-based polymers as biosorbents for the removal of pharmaceuticals should be evaluated.

## 6. Advantages, Challenges and Future Perspectives on Pharmaceutical Phycoremediation

The wastewater treatment industry is confronting challenges with enormous contaminant loads. Thus, the development of new, alternative wastewater treatment systems that incorporate eco-friendly and more profitable technologies is needed [312]. This review has emphasized the potential of exploiting algae for the treatment of contaminants, especially pharmaceutical effluents. The widespread use of phycoremediation in wastewater treatment plants may bring a revolution in the field of environmental conservation. Algae present interesting advantages, as they are fast-growing, can remove both pharmaceuticals and nutrients from wastewater, and the remaining biomass can be used as a valuable bioresource to produce biofuel or other high-value by-products. 

However, there remain considerable challenges to the commercialization of phycoremediation [126]. A study is required that concentrates on cost efficiency and the environmentally friendly aspects of algae mass production as a side product of utilizing urban wastewater or wastewater from livestock [312,313,314], since one of the biggest challenges in using microalgae in phycoremediation is biomass harvesting to obtain cell-free effluents. Most of the microalgae of commercial interest are microscopic in size and are evolutionally adapted to remain suspended in the water column. Due to their unicellular forms and low population density, commercial biomass harvesting of microalgae is difficult, and the cost of biomass recovery is usually significant [315,316] Moreover, the harvesting process strictly depends on microalgae characteristics, such as cell size and population density [317,318,319]. Harvesting technologies may involve one or more steps and incorporate different biological (bioflocculation and microalgae immobilization), physical (centrifugation, gravity sedimentation, filtration and dissolved air flotation) and chemical (chemoflocculation) processes, which have been extensively described in the literature [318,320,321], but most of them are energy-consuming, making phycoremediation less attractive compared to other remediation methods [322]. Thus, reducing the costs of biomass harvesting is a problem that is currently widely investigated using single- and multiple-step harvesting systems; however, none of those systems are ideal because numerous factors (algal species, culture system, culture volume, total biomass yield, etc.) need to be considered [323]. According to recent studies, the use of different harvesting methods applied sequentially (e.g., flocculation followed by membrane filtration and combined with centrifugation) seems to be a promising solution to reduce the costs of phycoremediation [323].

According to [171,324], a biotechnological approach can bring great benefits to the improvement of the efficiency of phycoremediation. Genetic engineering tools such as mutation breeding, hybridization, gene editing and domestication can significantly improve the process of phycoremediation. Genetically modified algae, equipped with new or increased capacities for degrading various compounds, will have an important future in this field, since such microorganisms will be able to effectively remove pharmaceuticals from wastewater; this has already been reported for the heavy-metal-binding transgenic alga, *Chlamydomonas reinhardtii* [325]. Databases are available in which the sequenced genomes of some microalgae have been presented [171,326], and this is a promising tool and perspective for creating algae varieties that perform better with the biosorption or biotransformation of pharmaceuticals. An interesting system for removing pharmaceutical impurities may be an algae consortium used with other microorganisms, such as bacteria or fungi [327].

Unfortunately, most studies on drug phycoremediation remain limited to single compounds in the laboratory and are performed with synthetic media, despite it being well-known that wastewaters are complex matrices. Extensive research on an industrial scale is still needed to understand the complexity of the processes, the dependence on physio-chemical and biological factors and the mechanisms involved to determine the exact requirements for algae growth as well as the efficiency and profitability of the process. The conducted research indicates enormous potential for algae in the treatment of wastewater from pharmaceuticals, as well as other pollutants whose toxic effects on non-target organisms are still intensifying. There is abundant space for further progress in determining the toxic mechanisms of pharmaceuticals in algae. Further research should be completed to investigate and determine how biosorption and biotransformation of selected drugs occur in algae. The development of this field also requires cooperation between academic institutions as well as research and development with the industry and government institutions. The final stage should focus on developing precise rules and guidelines governing the use of algae in treatment plants [328].

Therefore, the main directions for future research and perspectives of pharmaceutical phycoremediation should include (i) reducing the release of pharmaceuticals into water bodies; (ii) improving knowledge of the fate, effects and risks of pharmaceuticals in the environment, including mixtures and transformation products; (iii) improving sewage treatment by using new cost-efficient and eco-friendly technologies with algae to replace conventional wastewater treatment; and (iv) improving the biodegradability of pharmaceuticals and other wastewater contaminants [329].

## 7. Conclusions

Industrialization and urbanization in developed countries have led to increased contamination of water resources. Among anthropogenic contaminants such as heavy metals, polycyclic aromatic hydrocarbons and pesticides, special attention is currently paid to pharmaceuticals, which are classified as contaminants of emerging concern. These contaminants are potentially hazardous for non-target organisms and can pose a threat even for humans by reaching drinking water resources. Thus, efficient methods of wastewater treatment are urgently needed. Moreover, different chemical and physical conventional wastewater treatments are usually not efficient in removing emerging contaminants from wastewater; therefore, algae-based remediation methods are being widely investigated. Zero-waste technologies, where algal biomass is grown in wastewater, are promising due to their eco-friendliness, profitability and widespread availability.

Information obtained from the present review reveals the prevalence of pharmaceutical contaminants in the aquatic environment, their toxicity on non-target organisms and the potential advantages of phycoremediation over conventional wastewater treatments. This paper provides a comprehensive overview of different algal-based removal techniques, as well as the factors which may influence the removal efficiency of contaminants. This review of the literature clearly demonstrates both the challenges and advantages of phycoremediation. Thus, to be effective in remediating contaminants of emerging concern, including pharmaceuticals, intensive research on an industrial scale is still needed. This requires cooperation among academia, researchers, the industry and government institutions. Overall, the main subjects that need to be addressed in pharmaceutical remediation are (i) preventing pharmaceutical “leakage” into water bodies, (ii) increasing knowledge of the fate, effects and risks of pharmaceuticals in the environment and (iii) improving wastewater treatment using new, cost-efficient, zero-waste and eco-friendly technologies.

## Figures and Tables

**Table 1:** Occurrence of selected NSAIDs in surface water, wastewater and drinking/underground water in different locations.

Surface Water (ng/L)	Reference	Wastewater (Effluents (E)/Influents (I)) (ng/L)	Reference	Drinking Water (DW)/Underground Water (UW) (ng/L)	Reference	Area
**Ibuprofen**						
<0.3–56.0 3730.1 222.0 2.2 0.0−346.0 21.0–2796.0 0.9−115.8 0.1−0.6 524.0−17,600.0	[42] [43] [44] [45] [46] [47] [48] [49] [50]	31,250.0 0−4926.0 (I) 20,130.0 0−10,600.0 (E) 0.2−1.9	[43] [44] [46] [51] [49]	223.6 (UW)/599.0 (DW) ND−1.2 92 5850.0 <LOD−17.2 7.0−836.7	[43] [51] [51] [52] [53]	UK Poland Portugal Sweden Serbia North America China Taiwan Vietnam South Africa
**Diclofenac**						
<0.5–261.0 5401.5 241.0 1.7−3.6 0−324 17.0–42.0 ND−1.5 0.3−0.4 1010.0−10,200.0	[42] [43] [54] [45] [46] [47] [48] [49] [50]	40,570.2 0–269.0 (I) 1338.0 286.0 0.1−1.0	[43] [44] [46] [55] [49]	2770.0 (GW)/114.3 (DW) <LOD−2.4 2.1−33.2	[43] [52] [53]	UK Poland Portugal Sweden Serbia North America China Taiwan Vietnam South Africa
**Naproxen**						
<0.3–55.0 1091.9 178.0 0.2 0−74.2 22.0 3.5 0.1−0.4 59,300.0	[42] [43] [54] [45] [46] [47] [56] [49] [50]	551,960.0 8.8−1617.0 (I) 208.0 23,210.0 (I) 470.0 0.1−0.6	[43] [44] [46] [57] [55] [49]	21.0 (GW)/13.0 (DW) 27.6 44.0 <LOD−3.1 128.0	[43] [46] [58] [52] [53]	UK Poland Portugal Sweden Serbia North America China Taiwan Vietnam South Africa
**Ketoprofen**						
<0.5–4.0 132.2 0.3−89.0 0.3−1.3 1.4−54.5 509 <0.5−0.5 443.0−9220.0	[42] [43] [54] [45] [59] [60] [49] [50]	233,630.0 289.0−589.0 (I) 247.0 0.1−1.6	[43] [44] [46] [49]	731.8 (GW)/166.9 (DW) 0.09 16.0 (DW) 4.1 (GW) ND	[43] [61] [46] [62] [63]	UK Poland Portugal Sweden Serbia China Vietnam South Africa

<LOD = below limit of detection; ND = non detected.

**Table 2:** Toxic effects of selected NSAIDs on various non-target aquatic organisms, based on [2,35,72].

Compound	Tested Organisms (Taxonomic Group)		Tested Concentration	Exposure Time	Effect (Acute and Chronic)	Reference
**Plants**						
Ibuprofen	*Desmodeus subspicatus*	Chlorophyta	315.0 (mg/L)		Growth inhibition (EC_50_).	[73]
	*Lemna minor*	Tracheophyta	22.0 (mg/L)	7 d	Growth inhibition (EC_50_).	[73]
**Animals**						
Ibuprofen	*Cyprinus carpio*	Pisces	7.1 (mg/L)	12, 24, 48, 72, 96 h	Genotoxic effects: DNA damage (the intensity of the tail DNA relative to the head).	[74]
	*Cyprinus carpio*		1.5, 3.0, 4.5, 6.0, 7.5, 9.0, 11.5 (mg/L)	96 h	Teratogenic effect: higher mortality of oocytes and delay in hatching. Delay in embryo development and embryo malformations.	[75]
	*Danio rerio*		0.04, 0.2, 1.0, 5.0, 25.0 (mg/L)	56 h	Reproduction disruption: disruption of cardiac physiology of embryos.	[76]
	*Danio rerio*		0.000092 (mg/L)		Genotoxic effects: DNA fragmentation, apoptosis and genomic alterations.	[77]
	*Danio rerio*		10.0, 100.0, 1000.0 (mg/L)	14 d	Genotoxic effects: disruption of gonadotropin production. Increase in the transcription level of genes involved in the acceleration of gametogenesis, maturation of oocytes in females and spermatogenesis in males.	[78]
	*Oryzias latipes*		0.0001 (mg/L)	21 d	Genotoxic effects: influence of sex steroid hormones. Changes in the production of estradiol (E2). Endocrine-disrupting effect: significant increase in vitellogenin (VTG).	[79]
	*Oryzias latipes*		0.01, 0.1, 1.0, 10.0, 100.0, 1000.0 (mg/L)	132 d	Genotoxic effects: disruption of reproduction processes and early life stages. Reproduction disruption: delay in spawning.	[79]
	*Crassostrea gigas*	Molluscs	1.0, 100.0 (mg/L)	7 d	Gene expression disorder: differences in gene transcription in gill tissue. Significant upregulation of CYTP450 genes.	[80]
	*Dreissena polymorpha*		0.2, 1.0, 3.0 (mM)	1 h	Acute cytogenotoxic effect: irreversible DNA damage and decrease in LMS.	[81]
	*Dreissena polymorpha*		1.0, 9.0, 35.0 (nM)	96 h	Oxidative stress: increase in activity levels of SOD, CAT, GPx and GST.	[82]
	*Ruditapes philippinarum*		0.1, 5.0, 10.0, 50.0 (mg/L)	35 d	Acute cytogenotoxic effect: decrease in LMS in haemolymph.	[83]
	*Ruditapes philippinarum*		0.1, 5.0, 10.0, 50.0 (mg/L)	14 d	Oxidative stress: increase in GPx activity and LPO.	[84]
	*Ampelisca brevicornis*	Crustaceans	0.05, 0.5, 5.0, 50.0, 500.0 (ng/g)	10 d	Oxidative stress: significant increase in DBF, GST and GPX activity.	[85]
	*Daphnia magna*		2.9 (mg/L)	48, 96 h	Genotoxicity effect: DNA damage.	[86]
	*Daphnia magna*		20.0, 40.0, 80.0 (mg/L)	24 h	Endocrine disruption: deregulation of eicosanoid metabolism, the endocrine system and oogenesis.	[87]
	*Daphnia magna*		20.0, 40.0, 80.0 (mg/L)		Decrease in reproduction or complete reproduction inhibition.	[88]
	*Daphnia magna*		0.0005, 0.005, 0.05 (mg/L)	21 d, 6 h	Oxidative stress: the induction of antioxidant enzymes (GST, SOD and CAT). Reproduction disruption: significant decrease in the total number of broods per female, body length and intrinsic growth rate.	[89]
	*Hediste diversicolor*	Polychaeta	5.0, 500.0 (ng/g)		Genotoxic effect: DNA damage.	[90]
**Plants**						
Diclofenac	*Desmodeus subspicatus*	Chlorophyta	72.0 (mg/L)		Growth inhibition (EC_50_).	[73]
	*Dunaliella tertiolecta*		185.7 (mg/L)	96 h	Growth inhibition (EC_50_).	[55]
	*Pseudokirchneriella subcapitata*		20.0 (mg/L)	96 h	Growth retardation.	[65]
	*Scenedesmus vacuolatus*		23.0 (mg/L)		Inhibition of reproduction.	[91]
	*Lemna minor*	Tracheophyta	7.5 (mg/L)	7 d	Growth inhibition (EC_50_).	[73]
	*Polystichum setiferum*		0.0003 (mg/L)	48 h	Hormetic effects in mitochondrial activity in spores.	[92]
**Animals**						
Diclofenac	*Cirrhinus mrigala*	Pisces	0.001 (mg/L)	96 h	Oxidative stress: induction of enzymatic activity.	[93]
	*Cyprinus carpio*		0.001 (mg/L)	96 h	Alterations in hematological and biochemical activities.	[94]
	*Cyprinus carpio*		17.6 (mg/L)	12, 24, 48, 72, 96 h	Genotoxic effects: DNA damage (the intensity of the tail DNA relative to the head).	[74]
	*Cyprinus carpio*		1.25, 2.5 and 5.0 (mg/L)	21 d	Deformations: histopathological changes in gills, liver and kidney. Lesions included necrosis of epithelial cells.	[95]
	*Danio rerio* embryos		12.5 (mg/L)	48 h	Oxidative stress: deregulation of kinase activities. Metabolic disorders: deregulation of gluconeogenesis and lipid metabolism.	[96]
	*Hoplias malabaricus*		0.2, 2.0, 20.0 (mg/kg)		Metabolic disorders: interferences with metabolic pathways. Oxidative stress: increase in the activity of SOD, GPx and GSH.	[97]
	*Oncorhynchus mykiss*		0.005 (mg/L)	28 d	Deformations: renal lesions and alterations in the gills.	[98]
	*Oryzias latipes*		7.1, 37.0,78.0 (mg/L)	14 d	Morphological abnormalities.	[99]
	*Rhamdia quelen*		25.0 (mg/L)		Behavioral changes: respiratory disorders and loss of balance.	[100]
	*Rhamdia quelen*		0.2, 2.0, 20.0 (mg/L)	21 d	Oxidative stress: significant reduction in SOD activity, increase in activity of GSH and GST. Disruption of antioxidant defense systems in the liver.	[101]
	*Brachionus calyciflorus*	Rotatoria	25.0 (mg/L)	48 h	Reproduction retardation.	[65]
	*Dreissena polymorpha*	Molluscs	0.2, 0.5, 0.8 (mM)	1 h	Acute cytogenotoxic effect: significant DNA damage.	[81]
	*Dreissena polymorpha*		1000.0 (mg/L)	96 h	Oxidative stress: increase in GST activity, LPO expression and methallothioneins (MTs) alterations.	[102]
	*Dreissena polymorpha*		0.001 (mg/L)	96 h	Oxidative stress: high lipid peroxidation levels. Significant reduction in haemocyte viability.	[81,103]
	*Perna perna*		20.0, 200.0, 2000.0 (ng/L)	48, 96 h	Genotoxic effects: DNA damage. Significant decrease in LMS. Gene expression upregulation. COX inhibition in gill tissue.	[104]
	*Atyaephyra desmarestii*	Crustaceans	13.3, 70.6 (mg/L)	96 h	Metabolism disorder: decrease in respiration under reduced oxygen content.	[105]
	*Carcinus maenas*		0.00001, 0.0001 (mg/L)		Osmoregulation disturbances. Effect on haemolymph osmolality and osmolality capacity.	[106]
	*Ceriodaphnia dubia*		2.0 (mg/L)	7 d	Reproduction inhibition.	[65]
	*Daphnia magna*		32.0 (mg/L)	21 d	Oxidative stress.	[103]
	*Daphnia magna*		9.7 (mg/L)	48, 96 h	Genotoxicity effect: DNA damage.	[86]
	*Arenicola marina*	Polychaetes	From 0.6 to 842.0 (ng/L)		Reproduction disruption: decrease in swimming speed of sperm.	[107]
	*Hediste diversicolor*		0.5, 1.0, 2.0 (mg/L)	28 d	Gene expression upregulation: significant effect on the activity of GST enzymes.	[108]
**Plants**						
Naproxen	*Cymbella* sp.	Ochrophyta	102.8 (mg/L)	72 h	Growth inhibition (EC_50_).	[109]
	*Desmodeus subspicatus*	Chlorophyta	>320.0 (mg/L)		Growth inhibition (EC_50_).	[73]
	*Raphidocelis subcapitata*		0.0318 (mg/L)	72 h	Growth inhibition (EC_50_).	[110]
	*Scenedesmus quadricauda*		101.5 (mg/L)	72 h	Growth inhibition (EC_50_).	[109]
	*Scenedesmus subspicatus*		625.5 (mg/L)	48 h	Growth inhibition (EC_50_).	[70]
	*Lemna minor*	Tracheophyta	24.2 (mg/L)	7 d	Growth inhibition (EC_50_).	[70]
**Animals**						
Naproxen	*Danio rerio*	Pisces	1.0, 100.0 (mg/L)	14 d	Gene expression: upregulation of gene expression. Metabolism disorders: upregulation of the activity of GST by affecting glutathione S-transferase P2 (GST P2) mRNA in the intestine.	[111]
	*Oryzias latipes*		0.005, 0.05, 0.5, 5.0, 50.0 (mg/L)		Endocrine disruption: significant increase in the expression of VTG and E2 receptors genes. Reduction in conditions: decrease in the survival of juvenile animals.	[112]
	*Daphnia magna*	Crustaceans	46.7 (mg/L)	48 h	Immobilization (EC_50_).	[112]
	*Daphnia magna*		2.9 (mg/L)	48, 96 h	Genotoxicity effect: DNA damage. Oxidative stress: increase in enzyme activity (SOD, CAT and GPx).	[86]
	*Hyalella azteca*		76.6, 339.2 (mg/kg)	48 h	Genotoxicity effect: DNA damage. Oxidative stress: increase in SOD and CAT activity and decrease in GPX activity.	[113]
	*Moina macrocopa*		74.1 (mg/L)	48 h	Immobilization (EC_50_).	[112]
	*Elliptio complanata*	Molluscus	0.6 to 23.0 (mg/L)	24 h	Immunotoxic effects. Phagocytic activity, intracellular esterase activity, cell adherence and lipid peroxidation.	[114]
	*Hydra magnipapillata*	Cnidaria	LC_50_ 52.0, 45.0, 43.0 (mg/L)	24, 48, 72 h	Morphological changes: stimulation of the contraction of the body column and tentacles. Genotoxicity effect: DNA damage or instability.	[115]
**Plants**						
Ketoprofen	*Lemna minor*	Tracheophyta	0.2, 1.2, 6.0, 30.0 (mg/L)	4 d	Oxidative stress: alterations in enzyme activities (CAT, GSTs and CA).	[116]
**Animals**						
Ketoprofen	*Ceriodaphnia dubia*	Crustaceans	From 1.0 to 1000.0 (mg/L)		Chronic toxicity. Effects on reproduction at the highest concentration.	[117]
	*Daphnia magna*		0.2, 1.2, 6.0, 30.0 (mg/L)	4 d	Oxidative stress: alterations in enzyme activities (CAT, GSTs and CA).	[116]
	*Mytilus galloprovincialis*	Molluscus	0.0025 (mg/L)	14, 30, 60 d	Alterations in immunological parameters, genotoxic effects and modulation of lipid metabolism. Reduction in lysosomal membrane stability.	[118]
	*Planorbarius corneus*		100.0 (mg/g)	48 h	Antipyretic effect. Inhibition of symptoms of behavioral fever and influenced thermal preference.	[119]

**Table 5:** Algal cells with different immobilized carriers in wastewater treatment, based on [3,265].

**Carrier Used**	**Group of Carrier**	**Algae Species**	**Removed Contaminants**	**References**
Alginate	Organic carrier (natural polymers)	*Chlorella*	Ni, Zn, Cd	[277]
		*Pediastrum boryanum*	Cr (VI)	[278]
		*Chlorella vulgaris*	Cu, Ni	[279]
Alginate beads		*Chlorella emersonii*	Hg	[280]
		*Tetraselmis chui*	Cu, Cd	[9]
Alginate gel		*Isochrysis galbana*	Cr (III)	[281]
Alginate		*Dunaliella salina*	P	[282]
Alginate		*Chlorella vulgaris* *Chlamydomonas reinhardtii*	Pb	[3]
Alginate		*Scenedesmus intermedius*	N, P	[272]
Chitosan	Organic carrier (natural polymers)	*Scenedesmus* spp.	Nitrate, phosphate	[283]
Carrageenan beads	Organic carrier (natural polymers)	*Scenedesmus acutus* *Chlorella vulgaris*	Zn, Cd, Cr	[284]
Carrageenan		*Anabaena doliolum* *Chlorella vulgaris*	N, P	[285]
Polyurethane foam	Organic carrier (synthetic polymers)	*Scenedesmus acutus* *Chlorella vulgaris*	Zn, Cd, Cr	[284]
Carboxymethyl cellulose (CMC) beads	Organic carrier (synthetic polymers)	*Chlamydomonas reinhardtii*	U (VI)	[7]
Silica gel	Organic carrier (synthetic polymers)	*Chlorella vulgaris*	Hg	[286]
Glass beads	Inorganic carrier	*Aulosira fertilissima*	Ni, Cr	[287]
